# Comparative study of three common bile duct closure techniques after choledocholithotomy: safety and efficacy

**DOI:** 10.1007/s00423-022-02597-3

**Published:** 2022-07-04

**Authors:** Mohammed Ahmed Omar, Alaa Ahmed Redwan, Marwa Nasrelden Alansary

**Affiliations:** 1 General Surgery Department, Faculty of Medicine, South Valley University, Qena, Egypt; 2 General Surgery Department, Faculty of Medicine, Sohag University, Sohag, Egypt; 3 Anesthesia and Intensive Care Department, Qena Faculty of Medicine, South Valley University, Qena, Egypt

**Keywords:** Choledocholithiasis, T-tube, Primary repair, Biliary stenting

## Abstract

**Purpose:**

T-tube drainage, primary closure, and biliary stenting are the common bile duct closure methods. There is great debate on the optimal duct closure technique after common bile duct exploration. This study aimed to assess the safety and efficacy of the three commonest common bile duct closure methods after common bile duct exploration for common bile duct stone for future generalization.

**Methods:**

In this analysis, 211 patients with common bile duct stone underwent common bile duct exploration from January 2016 to December 2020. The patients were divided according to common bile duct closure techniques into three groups, including the T-tube drainage group (63 patients), primary duct closure group (61 patients), and antegrade biliary stenting group (87 patients).

**Results:**

The incidence of overall biliary complications and bile leak were statistically significantly lower in the biliary stenting group than in the other two groups. Also, hospital stays, drain carried time, return to normal activity, re-intervention, and re-admission rates were statistically significantly lower in the biliary stenting group than in the other two groups. There were no statistically significant differences regarding operative and choledochotomy time, retained and recurrent stone, stricture, biliary peritonitis, cholangitis, and the cost among the three groups.

**Conclusions:**

We state that the biliary stenting procedure should be the preferred first option for common bile duct closure after common bile duct exploration when compared with T-tube drainage and primary duct closure.

**Trial registration:**

ClinicalTrials.gov PRS (Approval No. NCT04264299).

## Introduction

Choledocholithiasis is the second most frequent complication of cholecystolithiasis with many critical complications [1]. It occurs in 5–20% of patients with cholecystolithiasis and approximately 10% of patients undergoing cholecystectomy [1, 2]. The optimal treatment for common bile duct stone (CBDS) is still debatable and unclear, and the available treatment options include common bile duct exploration (CBDE) or endoscopic stone extraction with endoscopic retrograde cholangiopancreatography (ERCP) [3, 4].

Open CBDE was the standard treatment. However, it still plays an important role in some hospitals and some situations where ERCP or laparoscopy are not available or failed [5]. Although ERCP is commonly used, it is associated with many postoperative complications [6]. Recently, with improved laparoscopic instrumentation and surgeon’s laparoscopic skills, laparoscopic CBDE is becoming commonplace and widely used worldwide [7].

In CBDE, the stones can be accessed through the cystic duct or direct choledochotomy. The trans-cystic duct approach is considered safe and feasible with unnecessary external biliary drainage. However, it is constrained by the size, number, and position of the stones, and the diameter and shape of the cystic duct [8]. Direct choledochotomy is the communal and preferable approach especially in cases of dilated CBD, common hepatic duct stones, abnormal cystic duct anatomy, and large stone [9].

One of the major issues and debates is the safe and successful choledochotomy closure techniques [10]. The commonest available options include repair with T-Tube drainage (TTD) [10], primary duct closure (PDC) [11], and repair over antegrade biliary stent (ABS) [3]. These approaches have distinct technical characteristics, necessitate different postoperative management, and are associated with distinct morbidity, so they should not be considered identical procedures [3].

TTD is the traditional surgical technique [12]. It has several advantages such as postoperative CBD decompression, trans-tubal cholangiography, prevention of stricture, and availability of retained CBDS extraction [13, 14]. However, it has several potential complications up to 10% of patients [15]. The most frequent complications are bile leak, tract infection, T-tube dislodgement, electrolyte and nutritional disturbances, cholangitis, or acute renal failure [10, 13]. It also causes patient discomfort and long-term pain, as well as increased hospital admissions and thus an economic burden to the country [16].

Consequently, PDC has been described in the literature to overcome these adverse consequences of the T-tube [17, 18]. It preserves CBD integrity and restores normal physiological function, reduces postoperative complications, shortens the length of hospital stay [19], and eliminates the need for T-tube drainage, which is critical for reducing postoperative pain and improving quality of life [20]. However, it has some potential complications as a potential bile leak and CBD stricture which may occur owing to papillary edema and insufficient bile duct expansion [21].

Consequently, ABS was used to minimize TTD and PDC-related complications. Papers showed that ABS is an effective and safe technique that prevents TTD-related complications [9, 22] and at the same time it reduces biliary pressure without causing bile loss [23]. However, the biliary stent has some potential complications as clogging, pancreatitis, migration proximally or distally, cholangitis, and perforation [24].

To the best of our knowledge, few studies have been reported comparing these three techniques after CBDE with conflicting results and great debate regarding their significant differences in morbidity and mortality [3, 4, 25]. There is no consensus till now about the optimal choledochotomy repair after CBDE [25]. Our study aimed to evaluate the efficacy and safety of TTD, PDC, and ABS techniques of CBD repair, to provide more evidence for selecting the optimal duct repair after choledocholithotomy.

## Materials and methods

### Patients

This was a randomized controlled trial conducted from January 2016 to December 2020 at two tertiary centers of hepatobiliary surgery. The study population consists of all consecutive patients who underwent CBDE for CBDS. CBDS was diagnosed preoperatively by clinical features, laboratory data, abdominal ultrasound, and magnetic resonance cholangiopancreatography, and intraoperatively by fluoroscopy-guided intraoperative cholangiogram and/or choledochoscope.

The inclusion criteria were patients with confirmed CBDS aged from 12 to 80 years, CBD diameter > 0.8 cm and < 2.5 cm, American Society of Anesthesiologists (ASA) score I–III, and agreement to complete the study. The exclusion criteria were patients with acute suppurative cholangitis, acute pancreatitis, intrahepatic bile duct stones, biliary neoplasm, biliary malformation, distal CBD stricture, trans-cystic stone extraction, and CBD exploration followed by bilio-enteric anastomosis.

**Fig. 1:**
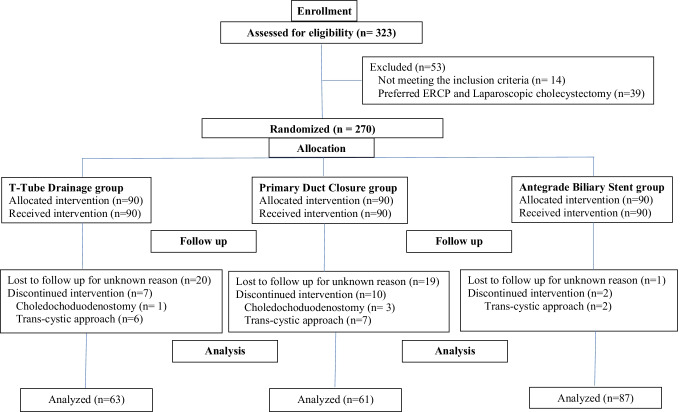
Flow chart of management

The number of patients needed was calculated. Considering a power of 80% and reliability of 0.05, we found that 59 patients should be present in each group. The study was started with a target of 323 patients for the possible loss of patients and data during the study and finally 211 were analyzed (Fig. 1).

The study protocol was approved by our hospitals’ ethical committee (Approval No. SVU 300) and was registered at *ClinicalTrials.gov PRS* (Approval No. NCT04264299). All participants or their relatives signed the written informed consent before recruitment.

### Operative techniques

All the operations were done by two experienced hepatobiliary surgeons. At the beginning of the study, open exploration was done routinely, and later with an increased learning curve, laparoscopic exploration has become more practiced. Antibiotic prophylaxis was given at induction. Supraduodenal longitudinal choledochotomy was performed adjusting its length to the size of the largest stone. The stones were extracted with a combination of saline irrigation, Dormia basket, or balloon extraction technique. Mechanical lithotripsy was used, if necessary. CBD clearance was confirmed by intraoperative cholangiogram, and/or choledochoscope.

In the TTD group, a latex rubber T-tube of appropriate size (10–16 Fr) was inserted in the choledochotomy incision after its fashioning and guttering. The choledochotomy incision was closed with an interrupted 4/0 vicryl suture^3^. In the PDC group, the choledochotomy was closed primarily with the previous suture. In the ABS group, a biliary plastic stent of appropriate size (8–10 Fr) and length (8–15 cm) was inserted in the choledochotomy and was passed throughout the papilla by gentle pushing. A 0.2 cc Fogarty balloon catheter or guidewire was inserted through the stent itself as a guide in difficult cases [26]. Trans-papillary stent placement was confirmed by choledochoscopy and/or duodenoscopy before choledochotomy closure with the previous suture.

In all patients with concomitant gall stones, cholecystectomy was performed at the end of the operation. Saline flushed through the T-tube or the trans-cystic cholangiography catheter to rule out leakage. A sub-hepatic drain was inserted in all patients for potential bile leak drainage. The anesthetist calculated the operative and choledochotomy closure time.

### Postoperative care and hospital discharge

The patients were started oral intake as tolerated. The patients were monitored regarding the vital signs, subhepatic drain and T-tube (if inserted) output, and liver function tests daily until discharge. An intravenous non-narcotic was used routinely twice daily. In severe uncontrollable pain, an opioid was added on demand. Postoperative pain was measured according to a visual analog scale (VAS) from 0 (no pain) to 10 (maximum pain) on the first three postoperative days (POD 1–3). The patients were discharged from the hospital 48–72 h postoperatively once there was no bile spillage in the drain, free abdominal US, and the sub-hepatic drain was removed. Otherwise, if the bile spillage was continued and the patient was asymptomatic, the patient was followed up at the outpatient clinic, and visits were scheduled accordingly until the spillage stopped and the drain can be safely removed.

### Follow-up

The patients were followed up at 2 weeks, 1, 6, and 12 months after discharge to exclude cholestasis. Each patient was evaluated clinically and with liver function tests and the abdominal US. T-tube drains were left open until a T-tube cholangiogram was done on the first follow-up visit. Once satisfactory cholangiography was obtained, the T-tube was removed. An abdominal X-ray was done for patients with a biliary stent on the second follow-up visit, and if the stent was still in place, it was removed endoscopically as an outpatient procedure on the same day.

### Data collection

The preoperative patient demographics and the intraoperative and postoperative characteristics were collected and analyzed. The postoperative biliary complications were classified according to Dindo–Clavien classification system [27].

### Outcome measures

The primary outcomes were postoperative biliary complications. The secondary outcomes were postoperative: pain severity, opioid requirement, TBIL level, hospital stays, drain carried time, cost, time to return to normal activity, re-intervention, readmission, and patient satisfaction.

### Statistical analysis

The data were analyzed in the statistical program SPSS 16.0 for Windows (SPSS, Chicago, IL, USA). Normal distribution data were confirmed with the Shapiro–Wilk test. The categorical data were expressed as frequencies and percentages and were compared with a chi-square test. Normally distributed data were expressed by the mean ± standard deviation (SD) and were compared with *t* test or analysis of variance. Statistical significance was determined as a *P* value of 0.05 or less.

## Results

From January 2016 to December 2020, 211 patients underwent successful CBDE through a choledochotomy approach. TTD was performed in 63 patients (29.9%), PDC was performed in 61 patients (28.9%), and ABS was performed in 87 patients (41.2%).

The baseline characteristics of all patients are listed in Table 1. There were no statistically significant differences among the 3 groups regarding the baseline and intraoperative characteristics (Tables 1 and 2). The causes for conversion were dense adhesions, unclear anatomy, uncontrolled bleeding, and failure to obtain a satisfactory CBD clearance. The intraoperative findings correlated with the preoperative radiological finding in all patients.

**Table 1:** Patients demographics and clinical characteristics

Parameters	TTD group (*n* = 63)	PDC group (*n* = 61)	ABS group (*n* = 87)	*P* value
Age (years)	41.3 ± 9.9	38.9 ± 7.9	41.9 ± 8.5	0.89
Sex (M/F)	24/39 (38.1/61.9)	25/36 (41/59)	30/57 (34.5/65.5)	0.21
BMI (kg/m^2^)	28.9 ± 4.7	29 ± 4.4	27.8 ± 5.2	0.87
Comorbidities	19 (30.1)	17 (27.9)	28 (32.2)	0.63
Previous abdominal surgery				
•Prior abdominal surgery	36 (57.1)	31 (50.8)	47 (54)	0.32
•Previous cholecystectomy	2 (3.2)	1 (1.6)	2 (2.3)	0.91
Symptoms				
•Abdominal pain	58 (92.1)	55 (90.2)	81 (93.1)	0.34
•Jaundice	52 (82.5)	51 (83.6)	71 (81.6)	0.63
•Acute cholecystitis	9 (14.3)	12 (19.7)	17 (19.5)	0.94
•Others	11 (17.5)	9 (14.7)	20 (23)	0.56
Liver functions tests				
•TBIL (mg/dl)	7.2 ± 2.9	7.3 ± 2.7	6.9 ± 2.6	0.13
•DBIL (mg/dl)	5.9 ± 2.3	5.8 ± 2.2	5.4 ± 1.9	0.59
•ALP (U/L)	423 ± 89.5	478 ± 105.3	401 ± 83.5	0.67
CBD diameter (mm)	12.7 ± 3.7	13.3 ± 3.5	13.1 ± 3.7	0.98
CBDS number				
•Single	12 (19)	12 (19.7)	21 (24.1)	0.76
•Multiple	51 (81)	49 (80.3)	66 (75.9)	0.85
CBDS size				
•Small (≤ 10 mm)	58 (92.1)	52 (85.2)	77 (88.5)	0.39
•Large (> 10 mm)	5 (7.9)	9 (14.8)	10 (11.5)	0.87
Concomitant gallstones	57 (90.5)	58 (95.1)	84 (96.5)	0.87
ASA score				
•ASA I	39 (61.9)	34 (55.7)	51 (58.6)	0.06
•ASA II	22 (34.9)	27 (44.3)	35 (40.2)	0.87
•ASA III	2 (3.2)	0 (0)	1 (1.2)	0.06

Results expressed as means ± SD, or the number of patients and percentage (%)

*TTD* T-tube drainage, *PDC* primary duct closure, *ABS* antegrade biliary stenting, *BMI* body mass index, *TBIL* total bilirubin, *DBIL* direct bilirubin, *ALP* alkaline phosphatase, *CBD* common bile duct, *CBDS* common bile duct stone, *ASA* American Society of Anesthesiology

**Table 2:** Intraoperative characteristics

Parameters	TTD group (*n* = 63)	PDC group (*n* = 61)	ABS group (*n* = 87)	*P* value
Approach				
•Laparoscopy	26 (41.3)	20 (32.8)	45 (51.7)	0.76
•Open surgery	31 (49.2)	30 (49.2)	35 (40.2)	0.54
•Conversion to open surgery	6 (9.5)	11 (18)	7 (8.1)	0.32
Complete CBD clearance	63 (100)	61 (100)	87 (100)	1
Operative time (min)	100.1 ± 27.4	95.3 ± 24.4	107.2 ± 31.15	0.06
Choledochotomy closure time (min)	13 ± 3.1	8 ± 1.9	15 ± 4.7	0.07
Blood loss (ml)	52 ± 7.9	60 ± 9	57 ± 6.4	0.89

Results expressed as means ± SD, or the number of patients and percentage (%)

*TTD* T-tube drainage, *PDC* primary duct closure, *ABS* antegrade biliary stenting, *CBD* common bile duct

Bile leak grade A was statistically significantly lower in the ABS group when compared with TTD and PDC groups. All patients with bile leak were managed conservatively except 3 patients in the PDC group (2 patients grade B were managed with ERCP and stent, and 1 patient grade C was managed with re-exploration and repair over a plastic stent). There were no statistically significant differences regarding retained and recurrent stone and stricture, and all these patients were managed endoscopically. In the TTD group, 6 patients developed specific complications after T-tube removal in the form of self-limited biliary fistula in 4 patients (6.3%), biliary peritonitis due to incomplete sinus tract formation in 1 patient (1.6%) who required laparoscopic re-exploration for lavage and drainage, and subhepatic collection in 1 patient (1.6%) who required percutaneous drainage. In ABS group, 1 patient (1.1%) developed migrated biliary stent and he was managed endoscopically (Table 3).

**Table 3:** Postoperative biliary complications *

Parameters	TTD group (*n* = 63)	PDC group (*n* = 61)	ABS group (*n* = 87)	*P* value
Bile leak	3 (4.8)	13 (21.3)	2 (2.3)	**0.001**
•*Grade A*	*2 (3.2)*	*9 (14.7)*	*2 (2.3)*	***0.01***
•*Grade B*	*1 (1.6)*	*3 (4.9)*	*0 (0)*	*0.63*
•*Grade C*	*0 (0)*	*1 (1.6)*	*0 (0)*	*0.87*
Recurrent CBDS	1 (1.6)	1 (1.6)	2 (4)	0.87
Biliary stricture	1 (1.6)	1 (1.6)	0 (0)	0.4
Biliary peritonitis	0 (0)	0 (0)	0 (0)	
Cholangitis	0 (0)	0 (0)	0 (0)	
Retained CBDS	1 (1.6)	0 (0)	0 (0)	0.9
Specific complications	6 (9.5)	0 (0)	1 (1.1)	
•*T-tube related complication*	*6 (9.5)*	*0 (0)*	*0 (0)*	
•*Stent related complication*	*0 (0)*	*0 (0)*	*1 (1.1)*	

Results expressed as the number of patients and percentage (%). *TTD* T-tube drainage, *PDC* primary duct closure, *ABS* antegrade biliary stenting, *CBDS* common bile duct stone. Bold entries are the significant results

The overall biliary complications were statistically significantly lower in the ABS group when compared with TTD and PDC groups. Only grades I and III biliary complications showed statistically significant differences (*P* = 0.01 and *P* = 0.01). There was no mortality directly associated with the surgical technique in any of the study groups (Table 4).

**Table 4:** Dindo classification of postoperative specific biliary complications

Grades	TTD group (*n* = 63)	PDC group (*n* = 61)	ABS group (*n* = 87)	*P* value
Grade I	6 (9.5)	9 (14.7)	2 (2.3)	**0.01**
*Bile leak grade A*	*2*	*9*	*2*	
*Biliary fistula*	*4*	*0*	*0*	
Grade II	1 (1.6)	1 (1.6)	0 (0)	0.09
*Bile leak grade B*	*1*	*1*	*0*	
Grade III	5 (7.9)	5 (8.2)	3 (3.4)	**0.01**
*Bile leak grade B*	*0*	*2*	*0*	
*Bile leak grade C*	*0*	*1*	*0*	
*Retained CBDS*	*1*	*0*	*0*	
*Recurrent CBDS*	*1*	*1*	*2*	
*CBD strictures*	*1*	*1*	*0*	
*Subhepatic collection*	*1*	*0*	*0*	
*Biliary peritonitis*	*1*	*0*	*0*	
*Migrated CBD stent*	*0*	*0*	*1*	
Grade IV	0	0	0	
Grade V	0	0	0	
Total	12 (19)	15 (24.6)	5 (5.7)	**0.001**

Results expressed as the number of patients and percentage (%)

*TTD* T-tube drainage, *PDC* primary duct closure, *ABS* antegrade biliary stenting, *CBDS* common bile duct stone, *CBD* common bile duct. Bold entries are the significant results

There was higher VAS (POD 1–3), opioid requirements, and less patient satisfaction in the TTD group when compared with the other 2 groups with a statistically significant difference. There was a statistically significant rapid reduction in the total bilirubin level in the POD3 and POD5 in the TTD group and ABS group while this significant difference disappears in the POD7 among all groups. Hospital stays, drain carried time, and return to normal activity were statistically significantly shorter in the ABS group when compared with the other 2 groups. Also, reintervention and readmission were statistically significantly lower in the ABS group when compared with TTD and PDC groups (Table 5).

**Table 5:** Postoperative characters

Parameters	TTD group (*n* = 63)	PDC group (*n* = 61)	ABS group (*n* = 87)	P_1_	P_2_	P_3_
VAS (POD 1–3)	6 ± 1.1	3 ± 0.7	3.2 ± 0.8	**0.01**	**0.01**	0.85
Patients required opioid	14 (22.2)	7 (11.5)	9 (10.3)	**0.01**	**0.02**	0.63
Postoperative TBIL (mg/dl)						
•POD 3	3.1 ± 1.4	4.8 ± 2.2	3.3 ± 1.5	**0.04**	0.39	**0.03**
•POD 5	1.1 ± 0.5	3.3 ± 1.3	1.9 ± 0.7	**0.01**	0.76	**0.01**
•POD 7	1.1 ± 0.5	1.4 ± 0.6	1.2 ± 0.5	0.98	0.86	0.67
Hospital stays (days)	3.9 ± 1.1	4.8 ± 1.9	2.9 ± 0.8	**0.03**	**0.01**	**0.02**
Drain-carried time (days)	3.8 ± 1.6	6.4 ± 2.8	2.5 ± 1.2	**0.04**	**0.001**	**0.01**
Cost of treatment (USD)						
•Index cost	945 ± 171	978 ± 193	870 ± 160	0.47	0.12	0.65
•Total cost	1070 ± 244	1218 ± 263	1030 ± 231	0.12	0.06	0.09
Return to normal activity (days)	18.4 ± 3.3	13.2 ± 2.9	8.6 ± 1.9	**0.01**	**0.001**	**0.01**
Re-intervention	5 (7.9)	5 (8.2)	2 (2.3)	0.96	**0.04**	**0.04**
•ERCP, sphincterotomy, and stone removal	2	1	2			
•ERCP, dilatation and stenting	1	3	0			
•Re-exploration and CBD repair over stent	0	1	0			
•Re-exploration and lavage	1	0	0			
•Percutaneous drainage	1	0	0			
Readmission (no.)	3 (4.7)	3 (4.9)	0 (0)	0.96	**0.04**	**0.04**
Patient satisfaction	2.6 ± 0.5	4.1 ± 0.4	4 ± 0.5	**0.01**	**0.01**	0.97

Results expressed as mean ± SD; number of patients and percentage (%); numbers. *TTD* T-tube drainage, *PDC* primary duct closure, *ABS* antegrade biliary stenting, *VAS* visual analogue score, *POD* postoperative day, *TBIL* total bilirubin, *USD* United States dollar, *ERCP* endoscopic retrograde cholangio-pancreatography, *CBD* common bile duct P_1_ represents the comparison between the PDC group and TTD group, P_2_ represents the comparison between the AGS group and TTD group, P_3_ represents the comparison between the PDC group and AGS group. Bold entries are the significant results

## Discussion

The commonest serious postoperative biliary complications of CBDE are bile leak and stricture [28]. The manipulation for stone removal can result in papillary spasm and edema which obstruct the proper bile drainage and results in biliary hypertension and bile leak throughout the choledochorraphy [3, 29]. However, the assumption of CBD drainage after choledochotomy to decrease biliary pressure is a matter of controversy [30].

To avoid this, choledochorraphy was traditionally done over a T-tube as a drainage method. But significant morbidity of T-tube was recorded both when the T-tube was in place or after its removal [31]. Many papers revealed that PDC of CBD is safe and feasible as a closure over T-tube [32, 33] with reported benefits in the form of decreased operative time and hospital stay [15, 20]. However, concerns about a higher incidence of biliary stricture after PDC have been raised [28]. Recently to circumvent this, a choledochorraphy was done over an ABS with a reported significant decrease in morbidity comparable with TTD and PDC [25, 34].

Recent papers revealed a great controversy with no strong consensus on which method is considered the perfect one for duct closure and gives the optimal outcome regarding biliary complications [4, 14, 25, 35]. To the best of our knowledge, few studies with conflicting results have been reported comparing these three techniques for CBD. Our study compared the efficacy and safety of TTD, PDC, and ABS techniques for choledochotomy closure.

Our results showed that the postoperative biliary complications were significantly lower in the ABS group than the other two groups. Our result to some extent is consistent with two published papers [11, 25] that revealed slightly lower stent group-specific biliary complications than T-tube and primary closure group with an insignificant difference. A recent systemic review [11] revealed that there was no significant difference in biliary-specific complications between TTD and PDC, and similarly between TTD and ABS. However, when the biliary-specific complications were analyzed individually, differences were apparent between the different techniques for CBD closure. Also, Mangla et al. [9] reported that there was no difference between the ABS group and the TTD group regarding the overall incidence of postoperative biliary complications.

Zhang et al. [13] stated that CBD drainage is necessary to overcome the sphincter of Oddi swelling and acute pyogenic cholangitis which can result in biliary hypertension and increased bile leak. We agreed with the opinion [23, 36] stated that PDC may be necessary to be combined with ABS to achieve optimal CBD decompression. Our results revealed a lower incidence of bile leak in the ABS group than the other two groups with a statistically significant difference. This significant difference was present only in the grade A. Our result was agreed with Parra-Membrives et al. [3] who showed that the incidence of bile leak was significantly higher in the PDC group than in the TTD group and the ABS group and the higher significant difference was present only in grade A. In the contrast, A recent systemic review [11] revealed that there was no significant difference in bile leak between TTD and PDC, and similarly between TTD and ABS. Also, a recent meta-analysis [14] and two studies [13, 25] revealed that there was no significant difference between the TTD group and the PDC group regarding bile leak. Another meta-analysis [37] showed a lower incidence of bile leak in the PDC group than the TTD group with an insignificant difference and a lower incidence of bile leak in the ABS group than the TTD group with a significant difference.

The risk factors for recurrent CBDS primarily include duodenal-biliary reflux, bile stasis, acute distal CBD angulation, sustained dilation of the biliary system, and abnormal location of the papillae [38, 39]. Choledochorraphy technique might be irrelevant to recurrent CBDS [4, 13, 37], and this was compatible with our finding that revealed a statistically insignificant difference among the 3 groups. A recent meta-analysis [37] showed an equal incidence of recurrent CBDS between the PDC group and the TTD group with an insignificant difference and a higher incidence of recurrent CBDS in the ABS group than the TTD group with an insignificant difference.

Biliary stricture is one of the major concerns for patients who have undergone CBDE. In most studies [40–42], the rate of the biliary stricture was very low. Our result was consistent with this finding as the overall incidence of the CBD stricture was 0.9%. The main risk factor for biliary stricture was the small CBD diameter. To prevent this, the optimal CBD diameter for a safe choledochotomy should be at least 8–10 mm [15, 43, 44]. Moreover, our result was consistent with many published studies [13, 14, 37] that revealed that there was no statistically significant difference for CBD stricture among the 3 groups. Therefore, choledochorraphy is a relevant risk factor for biliary stricture if it is done under a suitable CBD diameter [37].

Residual CBDS are not correlated to the choledochorraphy technique and are considered a management failure rather than postoperative biliary complications. Our result was consistent with other studies [18, 45] that demonstrated that the incidence of residual stones varies from 0 to 3.5%. Only one patient (1.6%) in the TTD group showed a retained stone in the trans-tubal cholangiogram. Therefore, the assumption that the T-tube provides easy percutaneous access for retained CBDS extraction is seldom necessary and even if there is retained CBDS after PDC or ABS, it can be removed by ERCP without re-exploration [45]. We agree with Deng et al. [14] that this low incidence results from mandatory intraoperative confirmation of CBD clearance by cholangiogram and choledochoscope. Our study revealed no statistically significant difference among the 3 groups, and this was consistent with several studies [9, 11, 13, 14, 41].

Our study revealed no biliary peritonitis or cholangitis developed among the 3 groups and this was consistent with Xiao et al. [25] In contrast, a recent systemic review with meta-analysis [11] revealed a lower rate of postoperative biliary peritonitis in the PDC group versus the TTD group with a statistically significant difference, and no difference between the ABS group and the TTD group for this outcome. Another recent meta-analysis [37] revealed a lower rate of postoperative biliary peritonitis in the PDC and ABS group versus the TTD group with a statistically significant difference.

Our study showed that the ABS group had significantly lower grades 1 and III postoperative biliary complications compared with the TTD group and PDC group. In contrast, our result was inconsistent with Parra-Membrives et al. [3], who showed the highest level of major complications (Dindo-Clavien ≥ 3) in the TTD group and the lowest level in the PDC group. A recent two meta-analyses showed that the PDC group had significantly lower postoperative complications compared with the TTD group [14, 37]. Moreover, a recent clinical trial by Wu et al. [35] and a retrospective study by Zhou et al. [41] revealed that no differences were found between the TTD group and PDC group.

Our results revealed no procedure-related mortality among the three groups, and this was consistent with several published studies [4, 14, 17, 25, 46, 47]. In the contrast, a recent systemic review with meta-analysis [11] revealed a slightly lower rate of postoperative mortality in the PDC group and ABS group versus the TTD group with no significant difference. The mean VAS of the first 3 postoperative days and the opioid requirement were significantly higher in the TTD group than the PDC group and ABS group. This may be attributed to the T-tube-related pain.

Stone manipulation results in papillary spasm and edema which impair proper bile drainage in the first postoperative days [3]. Our results showed that TTD and ABS promote the postoperative return of bilirubin level to normal value. The level of the postoperative TBIL was statistically significantly lower in the TTD group and the ABS group than in the PDC group on the third and fifth postoperative days, while no statistically significant difference among the 3 groups on the 7th postoperative day. The same results were reported by Xiao et al. [25]. In contrast, El Hanafy et al. [47] reported that there was no statistically significant reduction in the serum bilirubin level on the first or third postoperative days between the TTD group and PDC group.

Our study found that patients with the PDC had shorter operative and choledochotomy closure times than those with the TTD and ABS with an insignificant difference. Our results were consistent with several studies [9, 13, 25, 35, 41, 45]. The long operative time in the ABS group was attributed to the time used for confirmation of stent position in the duodenum which was very crucial to guard against specific stent complications. The distal end of the stent is passed through the papilla under direct choledoscopic vision. If failed backward pulling, this indicates optimal stent position and length with distal stent shelf arrested on the papilla. We use choledoscopic confirmation as a routine and in doubtful cases, we use duodenoscopic confirmation for the correct stent position. Also, the long operative time in the TTD group was attributed to the time used for the T-tube preparation, manipulation, and fixation. In contrast, a recent systemic review [11] showed that the operative time was statistically significantly longer in the TTD group versus the PDC group and ABS group. Also, two meta-analyses [14, 17] and a comparative study [48] showed that the operative time was statistically significantly longer in the TTD group than in the PDC group.

The postoperative hospital stay is an important concern, and a long stay is neither beneficial to the patient nor the healthcare provider [17]. Our result showed that the hospital stay was shorter in the ABS group than in the other two groups. The same result was reported in two recent studies [9, 25]. In contrast, our result was inconsistent with some studies [4, 13, 14, 17, 48] that revealed that hospital stay was statistically significantly lower in the PDC group when compared with the TTD group. Moreover, a recent systematic review [11] and a comparative study [3] showed that hospital stay was statistically significantly longer in the TTD than in the PDC and ABS, but the two latter were no statistical difference. The shorter hospital stay for the ABS group in our study may be attributed to many factors as less postoperative pain and analgesia requirement, less postoperative complications, faster return to normal bilirubin level, rapid return to normal activity, and the shorter drain carried time.

In our study, the incidence of bile leak was reflected on the drain carried time. It was statistically significantly shorter in the ABS group than in the TTD group and the PDC group. Our result was inconsistent with some studies [4, 10, 47, 49] that showed that the PDC was superior to the TTD regarding the drain carried time. To our knowledge, no published studies compared the ABS and the PDC regarding the drain carried time. This result may be explained by the low incidence of postoperative bile leak in the ABS group.

Two systemic reviews [11, 17] and two comparative studies [14, 45] showed a statistically significant less cost in the PDC versus the TTD. Our results showed that there was no statistically significant difference in the index and total cost among the 3 groups and the biliary stenting was the most cost-saving procedure. Our results were consistent with Xiao et al. [25]. Theoretically, the PDC procedure is a cost-saving as it saves the price of the T-tube and the biliary stent, the price of T-tube cholangiogram, and the price of T-tube and biliary stent removal but in practice, this saving is offset by the lower rate of biliary complications and shorter hospital stay in the ABS group.

The patients in the ABS group returned to normal activity about 5 days earlier than the PDC group and 10 days earlier than the TTD group. This may be attributed to less postoperative pain and analgesia requirement, less hospital admission, less drain carried time, in addition to the time needed for trans-tubal cholangiogram and T-tube removal. The result regarding TTD and PDC was consistent with the published studies [9, 17, 45].

The numbers of re-interventions and readmissions were statistically significantly lower in the ABS group than in the other two groups. Our result was inconsistent with Parra-Membrives et al. [3] who showed that reoperation was done in the 3 patients (5.8%) of the TTD group and 2 patients (3.4%) of the ABS group, and none of the patients in the PDC group and readmission was more frequent in the TTD group (9.6%) while only in 5.2% of the ABS group and absent in the PDC group (0%). A recent systemic review [11] revealed that there was no significant difference in the reintervention between the TTD and the PDC, and similarly between the TTD and the ABS.

Patient satisfaction was statistically significantly less in the TTD group than in the PDC group and the ABS group. This is understandable as the patient must carry the T-tube for at least 2 weeks before its removal which diminishes their quality of life.

Based on the evidence from this paper, the biliary stent is associated with less postoperative biliary complications, reduced hospital stays, decreased drain carried time, faster return to normal activity, and reduced re-intervention and readmission. In addition, it is associated with decreased postoperative pain and opioid analgesia requirements and a high patient satisfaction rate when compared with the TTD group. Moreover, another significant advantage of the biliary stent is the easier cannulation via ERCP, increasing the success rate of postoperative endoscopic retained or recurrent stone extraction from 80% to nearly 100% [50].

However, our study has one limitation which was the short follow-up duration that did not allow perfect long-term postoperative complications such as recurrent stones and biliary stricture follow-up.

## Conclusion

Biliary stenting procedure revealed better results when compared with TTD and PDC in terms of postoperative biliary complications, hospital stays, drain carried time, medical cost, and return to normal activity. We recommend the ABS procedure as the first option for CBD repair after CBDE.
